# Association Between Chronic Kidney Disease and Glaucoma: Results From the Lifelines Cohort Study and UK Biobank

**DOI:** 10.1167/iovs.66.15.17

**Published:** 2025-12-03

**Authors:** Wei Liu, Ruru Guo, Siqi Wang, Zekai Chen, Mengxue Song, Valeria Lo Faro, Wenbo Zhang, Nigus Gebrmedhin Asefa, Mark Eijgelsheim, Harold Snieder, Nomdo M. Jansonius

**Affiliations:** 1 Tianjin Key Laboratory of Retinal Functions and Diseases, Tianjin Branch of National Clinical Research Center for Ocular Disease, Eye Institute and School of Optometry, Tianjin Medical University Eye Hospital, Tianjin, People's Republic of China; 2 Department of Ophthalmology, University of Groningen, University Medical Center Groningen, Groningen, The Netherlands; 3 Department of Epidemiology, University of Groningen, University Medical Center Groningen, Groningen, The Netherlands; 4 Department of Immunology, Genetics and Pathology, Science for Life Laboratory, Uppsala University, Uppsala, Sweden; 5 Department of Critical Care, University of Groningen, University Medical Center Groningen, Groningen, The Netherlands; 6 Department of Internal Medicine, Division of Nephrology, University of Groningen, University Medical Center Groningen, Groningen, The Netherlands

**Keywords:** chronic kidney disease (CKD), glaucoma, estimated glomerular filtration rate (eGFR), polygenic risk scores (PRS)

## Abstract

**Purpose:**

Chronic kidney disease (CKD) and glaucoma are major health burdens, yet their phenotypic and genotypic relationships remain poorly understood. This population-based study aims to explore the phenotypic and genotypic relationship between CKD and glaucoma.

**Methods:**

In this cross-sectional study, European-descended individuals aged 55 years + from the Lifelines Cohort (*n* = 21,475; 906 CKD cases) and UK Biobank (*n* = 90,133; 3323 CKD cases) were analyzed. CKD was defined as an estimated glomerular filtration rate (eGFR) <60 mL/min/1.73 m². In Lifelines, glaucoma was defined using a previously established algorithm, which integrated self-reported glaucoma diagnosis and treatment with NEI-VFQ-25 scores. In the UK Biobank, primary open angle glaucoma cases were identified using a combination of self-reported diagnoses, treatment history, and International Classification of Diseases (ICD) codes. Logistic regression assessed the phenotypic association of CKD and eGFR with glaucoma, adjusting for demographic and clinical factors. Genetic analysis included linkage disequilibrium score regression (LDSR), polygenic risk scores (PRS), and Mendelian randomization (MR).

**Results:**

The prevalence of glaucoma was 8.2% in Lifelines and 5.5% in the UK Biobank. In Lifelines, no association between eGFR and glaucoma was found, but eGFR quintile 5 showed increased odds of glaucoma when excluding possible cases (odds ratio [OR] = 1.58, 95% confidence interval [CI] = 1.07–2.23, *P* = 0.02). In the UK Biobank, per 10 mL/min/1.73 m² eGFR increase was associated with higher odds of glaucoma (OR = 1.04, 95% CI = 1.02–1.07, *P* = 0.001), and quintile 5 exhibited increased odds regardless of whether all cases were included (OR = 1.14, 95% CI = 1.03–1.25, *P* = 0.01) or possible cases were excluded (OR = 1.13, 95% CI = 1.02–1.25, *P* = 0.02). No significant associations were found between CKD and glaucoma. LDSR showed no genetic correlations, but PRS indicated a significant association between glaucoma PRS and higher eGFR. MR revealed no causal relationship between eGFR and glaucoma.

**Conclusions:**

No association was found between CKD and glaucoma, but higher eGFR was associated with increased odds of glaucoma. These findings challenge the assumed link between low eGFR and glaucoma and highlight the need for further research into the underlying mechanisms and potential clinical implications of this association.

Glaucoma is a group of diseases characterized by optic disc cupping and corresponding visual field defects, which result from degeneration of retinal ganglion cells.^1^ Without proper treatment, glaucoma can lead to irreversible blindness. Primary open angle glaucoma (POAG) is the most common form of glaucoma in people from African, Hispanic, and European descent.^2^ The exact etiology and pathophysiology of POAG is not fully understood. Risk factors for POAG include increasing age, high intraocular pressure (IOP), myopia, a family history of glaucoma, use of steroid medication, and systemic diseases such as hypertension and diabetes.^3^ A systemic condition for which the relationship with POAG is controversial, is chronic kidney disease (CKD).

CKD is defined as a glomerular filtration rate (GFR) of less than 60 mL/min per 1.73 m^2^ of body surface area or a urinary albumin excretion greater than 30 mg/g that exists for longer than 3 months. Due to the substantial overlap in histology, physiology, and pathophysiology between the eyes and kidneys, it has been suggested that eye and kidney diseases, such as POAG and CKD, may be closely interlinked. However, the findings are inconsistent and the overall picture remains far from complete, as discussed in our recent review.^4^ For instance, the Beijing Eye Study^5^ and Singapore Malay Eye Study^6^ found no association between CKD and POAG, whereas an association was observed in the East Asians subgroup of a large consortium of multiple Asian population-based studies.^7^ Additionally, a relationship between lower estimated GFR (eGFR) and POAG was identified in the 2010 to 2011 Korea National Health and Nutrition Examination Survey.^8^ The disparities among these studies can potentially be attributed to differences in study populations and design. Analyzing extensive population-based datasets while carefully accounting for confounding factors may provide further insights into the connection between CKD and POAG, and shed light on the pathophysiology of both diseases. However, as far as we know, there have been no large, population-based, studies investigating the relationship between CKD and POAG in European populations.

Genetic factors are widely recognized as having a significant impact on POAG,^9^ and their influence has also been acknowledged in CKD.^10^ This highlights a genetic basis and heritable nature of both diseases. In our recent review,^4^ we hypothesized the possibility of shared genetic pathways underlying CKD and POAG. However, there have been no studies exploring the genetic association between CKD and POAG. Both CKD and POAG exhibit a complex inheritance pattern, and the effectiveness of single genetic variants in assessing risk may be limited. Linkage disequilibrium score regression (LDSR), based on genome-wide association study (GWAS) results, and polygenic risk scores (PRS) analysis, which captures genetic variants from all over the genome in a single score, can help to further elucidate the hypothesized underlying shared genetics between CKD and POAG. Moreover, Mendelian randomization (MR) analyses can explore potential causal relationships between CKD and POAG by using genetic variants as instrumental variables and minimizing confounding bias.^11^

In this study, we aimed to elucidate the phenotypic and genotypic relationship between CKD and POAG in populations from European descent. For this purpose, we studied the phenotypic and genotypic association between POAG and CKD and between POAG and eGFR in two large datasets, the Lifelines Cohort Study and UK Biobank.

## Methods

### Study Design and Samples

Lifelines is a multi-disciplinary prospective population-based cohort study examining in a unique three-generation design the health and health-related behaviors of 167,729 persons living in the North of the Netherlands. It uses a broad range of investigative procedures in assessing the biomedical, socio-demographic, behavioral, physical, and psychological factors which contribute to the health and disease of the general population, with a special focus on multi-morbidity and complex genetics. Baseline data were collected between 2006 and 2013, and follow-up visits are scheduled every 5 years for at least 30 years. A questionnaire on diagnosis and treatment of eye conditions, which included the National Eye Institute Visual Function Questionnaire-25 (NEI-VFQ-25) and specific questions targeting common eye diseases, was administered to all participants aged 18 years and older (*n* = 110,759) during the first follow-up visit between 2014 and 2017. Further information on design, cohort structure, and data collection approaches is fully described elsewhere.^12^^,^^13^ The Lifelines initiative has been made possible by subsidy from the Dutch Ministry of Health, Welfare and Sport, the Dutch Ministry of Economic Affairs, the University Medical Center Groningen (UMCG), Groningen University and the Provinces in the North of the Netherlands (Drenthe, Friesland, Groningen). The Lifelines data collection was approved by the ethics committee of the University Medical Center Groningen (no. 2007/152) and was conducted in accordance with the tenets of the Declaration of Helsinki. Written informed consent was obtained from all participants.

The UK Biobank is a large, prospective, population-based cohort study, involving over 500,000 individuals aged 37 to 73 years during the initial assessment period from 2006 to 2010. Following the provision of electronic informed consent, participants underwent a comprehensive touchscreen questionnaire, various physical measurements, and collection of biosamples. Between 2009 and 2010, a substudy focusing on eye and vision was conducted in approximately 175,000 participants to enhance the baseline dataset.^14^ The UK Biobank was approved by the National Health Service North West Multicentre Research Ethics Committee (06/MRE08/65) and the National Information Governance Board for Health and Social Care. This research was conducted under UK Biobank application number 76627 and conformed to the tenets of the Declaration of Helsinki. Comprehensive details about the UK Biobank, encompassing the complete study protocol and specific test procedures, can be accessed online (https://www.ukbiobank.ac.uk).

In both cohorts, we only extracted data from European participants aged 55 years and over because both CKD and POAG are more prevalent in elderly patients.^15^^,^^16^

### Glaucoma Phenotyping

In Lifelines, glaucoma was defined using a previously established algorithm, which integrated self-reported glaucoma diagnosis and treatment with NEI-VFQ-25 scores.^15^ Briefly, participants in Lifelines were categorized as definite, probable, or possible glaucoma cases, or as healthy participants, based on this algorithm. Those who reported incisional surgery for glaucoma were classified as definite glaucoma cases. These cases were also used to identify a glaucoma-specific complaints pattern within the NEI-VFQ-25. Those who self-reported glaucoma (including the use of IOP-lowering medication and a history of glaucoma laser treatment) together with a glaucoma-specific complaints pattern above a certain threshold (0.08), were classified as probable glaucoma cases. As described in our earlier publication, this threshold (0.08) was defined as the value that maximized the Youden’s J statistic (J = sensitivity + specificity – 1) on the receiver operating characteristic curve in our training population. This threshold represented the optimal balance between sensitivity (50.0%) and specificity (89.8%) for discriminating definite glaucoma cases from matched controls based on their NEI-VFQ-25 subscale scores.^15^ Those who either self-reported glaucoma or had a glaucoma-specific complaints pattern were classified as possible glaucoma cases. The algorithm was applied to participants in the first follow-up visit with available eye questionnaire data. In this study, unless otherwise stated, the term “glaucoma” refers to a combination of definite, probable, and possible cases. The proxy excluded participants with self-reported macular degeneration or (laser) surgery for diabetes or retinal detachment, aiming for primary glaucoma.

In the UK Biobank, participants were considered cases if they reported a diagnosis of glaucoma (from touchscreen questionnaires and/or verbal interview) or previous surgical or laser treatment for glaucoma in either eye, or carried an International Classification of Diseases (ICD) code for POAG (ICD 9th Revision [ICD-9] = 365.1; and ICD 10th Revision [ICD-10] = H40.1) in their linked hospital records at any point before and up to 1 year after the baseline assessment.^17^ In our analysis, participants carrying an ICD code for primary angle closure glaucoma (PACG; ICD-9 = 365.2 and ICD-10 = H40.2) and secondary glaucoma (ICD-9 = 365.3, 365.4, 365.5, and 365.6; and ICD-10 = H40.3, H40.4, H40.5, H40.6, and H42*) were excluded. The cases carrying an ICD code for glaucoma suspect (ICD-9 = 365.0 and ICD-10 = H40.0) were considered as possible cases.

### CKD Related Variables and Covariates

CKD related variables were measured during the baseline visit from both cohorts. CKD was defined as eGFR <60 mL/min/1.73 m^2^ using the Chronic Kidney Disease Epidemiology Collaboration creatinine equation in the complete sample. In the Lifelines subsample with available 24-hour urine collection, we expanded our analysis to include albuminuria-based definitions of CKD (e.g., albuminuria alone, or combined with reduced eGFR) as per Kidney Disease: Improving Global Outcomes (KDIGO) guidelines.^18^^,^^19^

In Lifelines, systolic and diastolic blood pressure were measured 10 times during a period of 10 minutes, using an automated Dinamap Monitor (GE Healthcare, Freiburg, Germany). The average of the final three readings was used for each blood pressure parameter. In the UK Biobank, blood pressure was measured on 2 consecutive occasions with a 1-minute interval using an Omron 705 IT electronic BP monitor (OMRON Healthcare). The mean of the first and second automated readings was used for data analysis. The definitions of hypertension and other covariates (centered age squared, body mass index, diabetes, hyperlipidemia, education level, and smoking status) are shown in Supplementary Table S1 in the supplement material.

### Genetic Data

In Lifelines, genotypic data collection occurred in two phases. The initial subset, consisting of 15,638 participants, underwent genotyping using the Illumina CytoSNP 12 version 2 array (Illumina, San Diego, CA, USA). The second subset, comprising 38,030 participants, was genotyped using the Infinium Global Screening Array (GSA) MultiEthnic Disease Version. Following quality control procedures, both datasets were imputed using the Haplotype Reference Consortium panel r1.1 (http://www.haplotype-reference-consortium.org) via the Sanger imputation service. Additional information regarding genotyping, quality control measures, and imputation processes within Lifelines can be found in previously published literature.^13^^,^^20^

In the UK Biobank, genetic data for 488,377 participants were collected using 2 genotyping arrays. The Affymetrix UK BiLEVE Axiom Array provided genotypes for 49,950 individuals, whereas the Affymetrix UK Biobank Axiom Array supplied genotypes for the remaining 438,427 individuals. Given that these arrays shared 95% of genetic markers, quality control measures and imputation were conducted jointly. Specifically, imputation utilized genetic architecture information obtained from the 1000 Genomes Project, the UK 10K, and the Haplotype Reference Consortium reference panels. Details of genotyping, imputation, and quality control have been described previously.^21^

### Linkage Disequilibrium Score Regression Analysis

The LDSR analysis was performed to estimate bivariate genetic correlations between CKD (traits) and glaucoma (traits), using the open source LDSC software version 1.0.1 (https://github.com/bulik/ldsc). We used the 1000 Genomes Project reference panel,^22^ specifically of European ancestry, for evaluating linkage disequilibrium (LD) between single nucleotide polymorphisms (SNPs). Our selection was focused on well-imputed HapMap3 SNPs. To ensure the analysis’s integrity, SNPs within the major histocompatibility complex region were omitted to prevent potential pleiotropic effects. Additionally, SNPs underwent further filtration, requiring a minimum minor allele frequency of >0.01 and an imputation score INFO >0.9 when such information was available in the summary dataset.

We utilized the GWAS summary statistics of European ancestry from the CKDGen consortium for CKD (*n* = 480,698) and eGFR (*n* = 567,460), both of which encompassed Lifelines, but excluded the UK Biobank. We used the GWAS summary statistics of European ancestry derived from a large-scale meta-analysis of POAG GWASs (*n* = 1,451,742), including both Lifelines and the UK Biobank, as outcome data. Additionally, we included GWAS summary statistics for IOP (*n* = 139,555) in our LDSR analysis. The detailed GWAS summary data used for LDSR is outlined in Supplementary Table S2.

### Polygenic Risk Scores Calculations

In this study, the SBayesRC method was applied to calculate PRS, which integrates GWAS summary statistics with functional genomic annotations to improve polygenic prediction of complex traits.^23^ It allows for fitting all imputed SNPs jointly, and considers differences between annotations in both the proportion of causal variants and the magnitude of causal effect sizes.

The GWAS summary statistics utilized for calculating the PRS of CKD and eGFR in the UK Biobank were identical to those used in the LDSR analysis. From these CKD and eGFR GWAS summary statistics, we first extracted the Lifelines Cohort effect prior to PRS calculation within the Lifelines Cohort using the R-package “Metasubtract.”^24^ Furthermore, we left out the Lifelines Cohort and UK Biobank, respectively, and recalculated the POAG GWAS summary statistics used in Lo Faro et al.^25^ (see Supplementary Table S2) prior to the PRS calculation of POAG within each cohort. We utilized the provided genomic annotation data and LD reference of Europeans from the UK Biobank. The PRS was centered at 0 with a standard deviation (SD) of 1 before analysis.

### Two-Sample Mendelian Randomization Analysis

We conducted MR analyses to explore a potential causal effect of eGFR on POAG. The analyses were performed in R software 4.0.3 (R Foundation for Statistical Computing, Vienna, Austria) using the TwoSampleMR R package (https://mrcieu.github.io/TwoSampleMR/). Genome-wide significant SNPs (*P* < 5 × 10^−8^) were selected as instrumental variables from GWAS data on eGFR (exposure in forward MR) and POAG (exposure in reverse MR; see Supplementary Table S2), and were further clumped with LD *r*^2^ < 0.001 within windows of 10 Mb. The random-effect inverse variance-weighted (IVW) method was used as the primary approach to estimate the causal effect, with statistical significance set at *P* < 0.05. Additionally, MR Egger and weighted median methods were used to assess the potential impact of pleiotropy on the causal estimates. Heterogeneity was assessed using Cochran’s Q statistics in both the MR Egger and IVW methods. The intercept term in the MR Egger regression model served as an indicator of whether horizontal pleiotropy influenced the results of the MR analysis. The detailed MR analysis procedure that we applied has been described previously.^11^

A reverse MR, which explored the causal effects of POAG on eGFR, was also performed to determine whether there was a reverse causal relationship.

### Statistical Analysis

Normally distributed continuous variables were described using mean and SD. Skewed continuous variables were described using median and interquartile range (IQR). Categorical variables were described as the number of samples (percentage). Logistic regression analysis was used for dichotomous outcomes, whereas linear regression analysis was used for continuous outcomes. The analyses for the association between kidney function (eGFR) and glaucoma were performed across the entire range in the full study population in both Lifelines and UK Biobank cohorts. Continuous predictors (eGFR and the PRSs for CKD, eGFR, and POAG) were also modeled as quintiles to account for potential nonlinear effects. The analyses were first performed for all glaucoma cases and then after excluding the possible cases, as it was less certain that these cases truly had glaucoma. A supplementary analysis stratified by diabetes status was also performed.

A directed acyclic graph was drawn using DAGitty version 3.0 (http://dagitty.net/dags.html; Supplementary Fig. S1) to determine the minimally sufficient adjustment sets. Two models, with varying degrees of covariate adjustment, were applied in this study. In model 1, we adjusted for age, centered age squared, and gender. In model 2, we adjusted for the minimally sufficient adjustment set of model 1 and additionally included body mass index, hypertension, diabetes, hyperlipidemia, education level, and smoking status. In the regression analysis involving PRS, the same model comprising age, centered age squared, gender, and the first 10 principal components was used in the UK Biobank, whereas we additionally adjusted for SNP array (CytoSNP or GSA) in Lifelines.

Data reorganization and analyses were performed in R software 4.0.3. *P* < 0.05 was considered statistically significant.

## Results

### Participants’ Baseline Characteristics

In total, our study included 21,475 participants from Lifelines and 90,133 participants from the UK Biobank for phenotypic analysis (Supplementary Fig. S2). Within Lifelines, there were 58 (0.27%) definite cases of glaucoma, 226 (1.05%) probable cases, and 1477 (6.88%) possible cases. The UK Biobank comprised 4954 cases of glaucoma (5.50%), including 449 possible cases (0.50%). Table 1 summarizes the characteristics of the study population categorized by glaucoma status in both Lifelines and the UK Biobank. Participants with glaucoma in Lifelines tended to be more often female subjects, whereas in the UK Biobank, they were slightly more often male subjects. Additionally, individuals with glaucoma were generally older, more likely to be obese, and had lower educational attainment across both cohorts. Furthermore, they exhibited a higher prevalence of hypertension, diabetes, and hyperlipidemia. Glaucoma cases also showed lower eGFR compared with controls, with a higher prevalence of CKD observed among glaucoma cases in both cohorts.

**Table 1.** Baseline Characteristics Stratified by Glaucoma Status in the Lifelines Cohort and UK Biobank

	Lifelines Cohort (*n* = 21,475)			UK Biobank (*n* = 90,133)		
Variables	All Glaucoma Cases (*n* = 1,761)	Possible Cases Excluded (*n* = 284)	Controls (*n* = 19,714)	All Glaucoma Cases (*n* = 4,954)	Possible Cases Excluded (*n* = 4,505)	Controls (*n* = 85,179)
Age, y	64.6 ± 6.3	65.9 ± 6.8	63.0 ± 5.9	63.4 ± 3.9	63.4 ± 3.9	62.3 ± 4.0
Gender, female	1,058 (60.1%)	165 (58.1%)	10,840 (55.0%)	2,290 (46.2%)	2,066 (45.9%)	46,034 (54.0%)
BMI, kg/m^2^	27.1 ± 4.1	26.9 ± 4.1	26.7 ± 3.9	27.8 ± 4.7	27.8 ± 4.7	27.6 ± 4.7
eGFR, mL/min/1.73 m^2^	82.0 ± 12.7	81.6 ± 13.6	83.1 ± 12.2	85.7 ± 12.8	85.7 ± 12.7	86.0 ± 12.6
CKD	112 (6.4%)	18 (6.3%)	794 (4.0%)	210 (4.2%)	189 (4.2%)	3,113 (3.7%)
Hypertension	1,022 (58.0%)	168 (59.2%)	10,345 (52.5%)	3,290 (66.4%)	2,987 (66.3%)	51,089 (60.0%)
Diabetes	200 (11.4%)	40 (14.1%)	1,437 (7.3%)	625 (12.6%)	572 (12.7%)	7,976 (9.4%)
Hyperlipidemia	817 (46.4%)	130 (45.8%)	8,624 (43.7%)	2,458 (49.6%)	2,239 (49.7%)	41,429 (48.6%)
Education level						
High	370 (21.0%)	49 (17.2%)	4,594 (23.3%)	1,326 (26.8%)	1,203 (26.7%)	25,209 (29.6%)
Middle	401 (22.8%)	78 (27.5%)	4,719 (23.9%)	1,513 (30.5%)	1,372 (30.5%)	26,720 (31.4%)
Low	990 (56.2%)	157 (55.3%)	10,401 (52.8%)	2,115 (42.7%)	1,930 (42.8%)	33,250 (39.0%)
Current or ever smoker	1,137 (64.6%)	189 (66.5%)	12,975 (65.8%)	2,472 (49.9%)	2,247 (49.9%)	40,749 (47.8%)

BMI, body mass index; CKD, chronic kidney disease (eGFR <60 mL/min/1.73 m^2^); eGFR, estimated glomerular filtration rate.

### Associations of Estimated Glomerular Filtration Rate and Chronic Kidney Disease With Glaucoma

**Figure 1.**
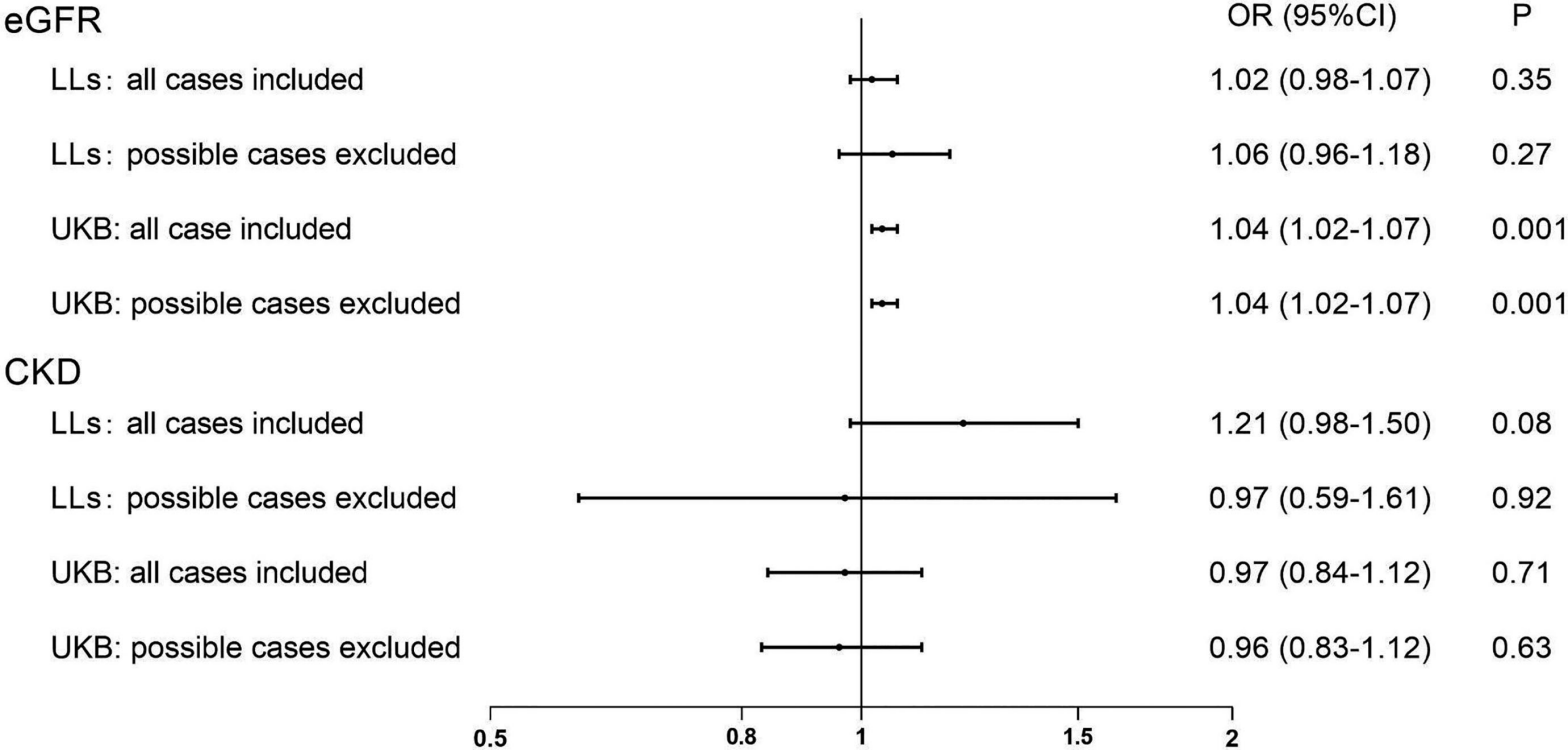
Associations of estimated glomerular filtration rate (per 10 mL/min/1.73m^2^ increase) and chronic kidney disease (eGFR <60 mL/min/1.73 m^2^) with glaucoma after full adjustment (model 2). eGFR, estimated glomerular filtration rate; CKD, chronic kidney disease; LLs, Lifelines Cohort; UKB, UK Biobank.

In Lifelines, no association of eGFR with glaucoma was found, regardless of whether all cases were included or possible cases were excluded (Fig. 1; Supplementary Table S3). When all cases were included, a significant association between CKD (defined as eGFR <60 mL/min/1.73 m^2^) and glaucoma was found in model 1 (odds ratio [OR] = 1.27, 95% confidence interval [CI] = 1.02–1.57, *P* = 0.03), but this association disappeared in model 2 (OR = 1.21, 95% CI = 0.98–1.50, *P* = 0.08). Upon excluding possible glaucoma cases, no associations of CKD with glaucoma were found in either model (see Fig. 1; Supplementary Table S3). Similarly, in the Lifelines subsample with albuminuria data, none of the additional CKD definitions that incorporated albuminuria were associated with glaucoma in any model (see Supplementary Table S3).

In the UK Biobank, we observed that eGFR (per 10 mL/min/1.73 m² increase) was significantly associated with glaucoma in the overall sample (OR = 1.04, 95% CI = 1.02–1.07, *P* = 0.001 in both models), and this result did not change much when excluding the 449 possible cases (OR = 1.04, 95% CI = 1.01–1.07, *P* = 0.002 in model 1; and OR = 1.04, 95% CI = 1.02–1.07, *P* = 0.001 in model 2; see Fig. 1; Supplementary Table S3), indicating that a higher eGFR level was linked to a higher odds of glaucoma. No significant associations were found between CKD and glaucoma.

### Associations of Estimated Glomerular Filtration Rate in Quintiles With Glaucoma

**Figure 2.**
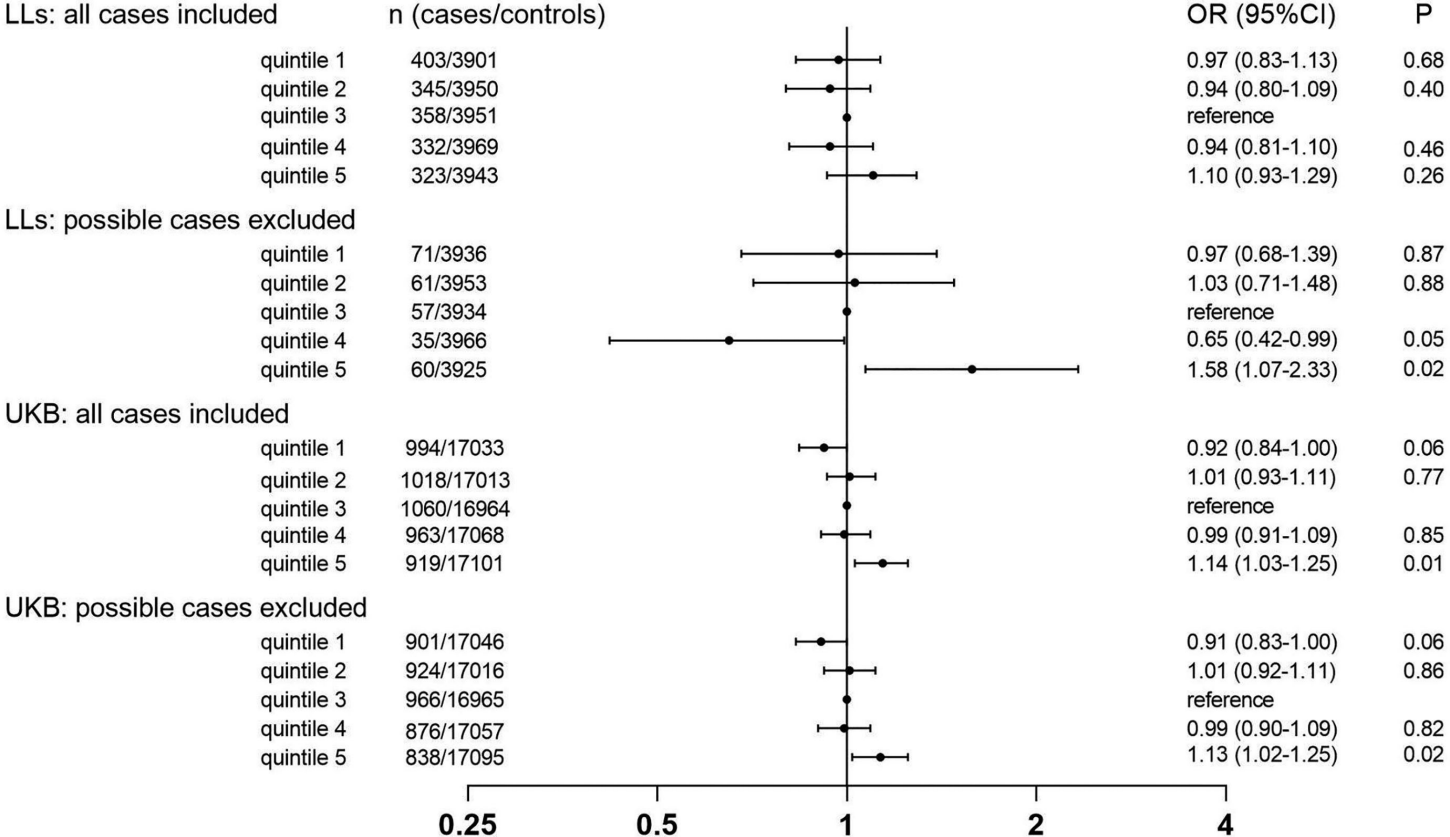
Odds ratios of glaucoma in estimated glomerular filtration rate quintiles with quintile 3 as reference after full adjustment (model 2). LLs, Lifelines Cohort; UKB, UK Biobank.

To capture potential nonlinear effects, we conducted an association analysis of eGFR in quintiles with glaucoma (Fig. 2; Supplementary Table S4). In Lifelines, when setting the middle eGFR quintile as the reference, there were no differences in the ORs of glaucoma across different eGFR quintiles when all cases were included. However, quintile 5 showed a significantly increased odds of glaucoma when possible cases were excluded (OR = 1.58, 95% CI = 1.07–2.23, *P* = 0.02 in model 2). Similarly, in the UK Biobank, with the middle eGFR quintile as the reference, quintile 5 also exhibited a significantly increased OR of glaucoma, regardless whether all cases were included (OR = 1.14, 95% CI = 1.03–1.25, *P* = 0.01 in model 2) or possible cases were excluded (OR = 1.13, 95% CI = 1.02–1.25, *P* = 0.02 in model 2).

Similar results were observed when the analysis was extended to participants aged 40 years and above, consistently showing no association with CKD but a positive association with higher eGFR.

### Diabetes Stratification Analyses

Among participants with diabetes, no significant associations were observed between glaucoma and eGFR or CKD in either the Lifelines or UK Biobank cohorts (Supplementary Table S5). Similarly, analysis of eGFR quintiles showed no significant associations in the UK Biobank. In Lifelines, whereas no association was found when all cases were included, the highest eGFR quintile showed increased odds of glaucoma compared to the middle quintile after excluding possible cases; it should be noted, however, that this analysis was based on a relatively small sample size (Supplementary Table S6).

Among participants without diabetes, no associations were observed between glaucoma and eGFR or CKD in the Lifelines cohort. In contrast, the UK Biobank data showed a significant association between higher eGFR levels and glaucoma, although no association was found with CKD (Supplementary Table S7). In the analysis of eGFR quintiles, no associations were detected in Lifelines, whereas in the UK Biobank, the highest eGFR quintile was associated with significantly increased odds of glaucoma compared to the middle quintile (Supplementary Table S8).

### Linkage Disequilibrium Score Regression Analysis

**Figure 3.**
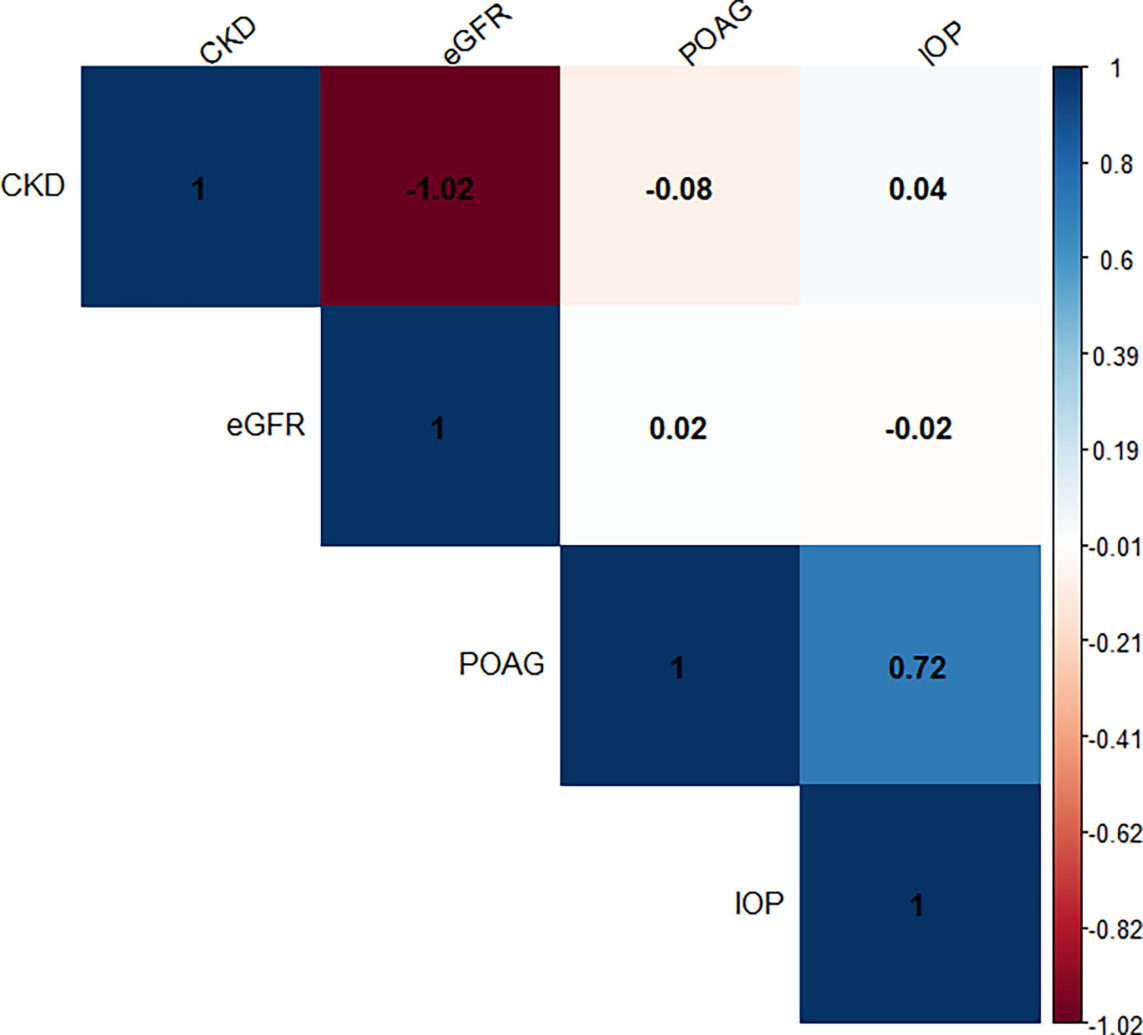
Genetic correlations between chronic kidney disease (traits) and glaucoma (traits). CKD, chronic kidney disease; eGFR, estimated glomerular filtration rate; IOP, intraocular pressure; POAG, primary open angle glaucoma. All *P* > 0.05 of POAG and IOP with CKD and eGFR.

No significant genetic correlation was found for POAG with CKD or eGFR; similarly, there was no significant genetic correlation for IOP with CKD or eGFR (all *P* > 0.05; Fig. 3).

### Polygenic Risk Scores Analysis

In this study, 11,628 participants from Lifelines and 88,622 participants from the UK Biobank with available genetic data were used for PRS analysis (see Supplementary Fig. S2). Our results showed statistically significant associations for CKD, eGFR, and glaucoma with their respective PRS in both cohorts (all *P* < 2 × 10^-16^; Supplementary Table S9), indicating a good predictive performance of SBayesRC generated PRSs for these traits. The variances explained by PRS for eGFR, CKD, and glaucoma were 14.6%, 4.9%, and 1.3% in Lifelines, and 9.7%, 1.8%, and 2.4% in the UK Biobank, respectively.

### Polygenic Risk Scores Analysis Between Chronic Kidney Disease and Glaucoma

Our study did not find any association of the CKD PRS with glaucoma in either cohort (Supplementary Table S10), nor were there differences in the ORs of glaucoma across different CKD PRS quintiles (Supplementary Table S11). Similarly, we found no association of the glaucoma PRS with CKD in either Lifelines (OR = 1.00, 95% CI = 0.91–1.10, *P* = 0.95) or the UK Biobank (OR = 1.00, 95% CI = 0.97–1.04, *P* = 0.98). Furthermore, there were no differences in the ORs of CKD across different glaucoma PRS quintiles (Supplementary Table S12).

### Polygenic Risk Scores Analysis Between Estimated Glomerular Filtration Rate and Glaucoma

Our study found no association of eGFR PRS with glaucoma in either cohort (Supplementary Table S13). Additionally, the ORs for glaucoma did not vary across different eGFR PRS quintiles (Supplementary Table S14). However, we found a significant positive association of glaucoma PRS with eGFR in the UK Biobank (β = 0.15, 95% CI = 0.07 to 0.23, *P* = 0.0002), with similar effect size in Lifelines although it was only borderline significant (β = 0.20, 95% CI = –0.01 to 0.43, *P* = 0.06). These results were confirmed in the quintile analysis. In Lifelines, glaucoma PRS quintile 5, and in the UK Biobank, glaucoma PRS quintiles 2, 3, 4, and 5, all showed statistically significantly higher eGFR compared to quintile 1 (Table 2).

**Table 2.** Associations of Glaucoma Polygenic Risk Scores (PRS) Quintiles With Estimated Glomerular Filtration Rate (eGFR) Compared to the First Quintile

Glaucoma PRS Quintiles	b	95% CI	*P* Value
Lifelines			
Quintile 1	Reference		
Quintile 2	0.22	−0.43 to 0.86	0.51
Quintile 3	−0.04	−0.69 to 0.60	0.89
Quintile 4	0.12	−0.52 to 0.76	0.72
Quintile 5	0.69	0.05 to 1.34	0.04
UK Biobank			
Quintile 1	Reference		
Quintile 2	0.35	0.10 to 0.60	0.006
Quintile 3	0.22	−0.04 to 0.47	0.09
Quintile 4	0.35	0.10 to 0.60	0.006
Quintile 5	0.49	0.24 to 0.74	0.0001

Models were adjusted for age, age², gender, and the first 10 genetic principal components in the UK Biobank, with additional adjustment for genotyping array (CytoSNP or GSA) in Lifelines.

### Two-Sample Mendelian Randomization Analysis

To explore the potential causal relationship between eGFR and glaucoma, we conducted a two-sample MR analysis. A total of 152 independent SNPs (*P* < 5 × 10^−8^) were used as proxies for eGFR. The analysis did not reveal statistically significant causal effects of genetically predicted eGFR on glaucoma. Significant heterogeneity was observed, suggesting potential pleiotropy, although there was little evidence of directional pleiotropy (Supplementary Table S15).

We also performed MR analysis to assess causality in the opposite direction. A total of 76 independent SNPs were used as proxies for glaucoma. Similarly, the analysis did not provide any evidence for a causal effect of glaucoma on eGFR (see Supplementary Table S15). Significant heterogeneity was again observed, but also here there was little evidence for directional pleiotropy.

## Discussion

In this study, we aimed to elucidate the relationship between CKD and glaucoma using both phenotypic and genetic data from two large population-based biobanks. Our results showed no association between CKD and glaucoma after adjusting for a wide range of potential confounders. Our LDSR analysis found no genetic associations of glaucoma with CKD or eGFR. However, the PRS analysis indicated a significant association of the glaucoma PRS with higher eGFR, although this was not confirmed by MR.

To date, the association between CKD and POAG has been equivocal and inconclusive. Most current literature reports no association between POAG and CKD, with only a few studies in East Asian populations indicating an association between POAG and CKD or lower eGFR.^26^ In our study, within the European population, we found no evidence of an association between glaucoma and CKD or lower eGFR. This null finding is in agreement with the results from a recent Swedish cohort study,^27^ which found no statistically significant increase in glaucoma risk associated with CKD. Although we observed a statistical association with higher eGFR in some analyses, this finding requires replication and further investigation before any firm conclusions can be drawn. Higher eGFR, often indicative of glomerular hyperfiltration, can result from afferent arteriolar vasodilation and/or efferent arteriolar vasoconstriction.^28^ This condition is not uncommon and is frequently observed in specific physiological contexts, such as following high protein intake or during pregnancy, and more commonly in patients with cardiometabolic risk factors like obesity, metabolic syndrome, or early-stage diabetic kidney disease.^29^ It is crucial to note that glomerular hyperfiltration is increasingly recognized not as a benign finding but as a pathophysiological state mechanistically linked to subsequent renal damage. We hypothesize that the association between glaucoma and higher eGFR might be explained by several, non-mutually exclusive pathways. First, it is possible that glomerular hyperfiltration could be linked to glaucoma through its association with arterial stiffness. Glomerular hyperfiltration could interact with abnormal metabolism to significantly enhance arterial stiffness.^30^ In line with this, glomerular hyperfiltration has been suggested as an early marker of arterial stiffness.^31^ Furthermore, arterial stiffness has been reported to be associated with an increased risk of glaucoma.^32^ Therefore, glomerular hyperfiltration may contribute to the development of glaucoma through its relationship with arterial stiffness. Second, the renin-angiotensin-aldosterone system (RAAS) represents another plausible mechanistic link. RAAS activation has been associated with glomerular hyperfiltration, primarily through elevated levels of angiotensin II, which causes efferent arteriolar vasoconstriction.^33^ Moreover, RAAS is thought to play a role in the pathogenesis of glaucoma. In human nonpigmented ciliary body epithelial cells, RAAS components have been shown to regulate the formation and secretion of aqueous humor. Angiotensin II has also been found to decrease trabecular outflow by stimulating cellular proliferation and increasing collagen deposition in the trabecular meshwork. Together, these findings suggest that the ocular RAAS is involved in both the production and drainage of aqueous humor and plays a key role in regulating IOP.^4^ Therefore, RAAS activation might simultaneously drive renal hyperfiltration and influence ocular pathophysiology. Third, a shared genetic predisposition is another intriguing possibility. Our recent large-scale meta-analysis of POAG GWAS identified several genes related to primary cilia that are implicated in the pathophysiology of glaucoma.^25^ In the kidneys, proper ciliary function is essential for regulating various cellular processes and maintaining tissue homeostasis. Disrupted ciliogenesis in renal tubular epithelial cells can contribute to persistent glomerular hyperfiltration by affecting the lumen of the renal tubules.^34^ This raises the hypothesis that pleiotropic genetic effects on ciliary function could predispose individuals to both glomerular hyperfiltration and glaucoma. These proposed mechanisms remain speculative and are intended to generate hypotheses for future research. The exact biological pathways, if any, linking higher eGFR to glaucoma require rigorous investigation in dedicated experimental and longitudinal studies.

To investigate the potential shared genetic pathways between CKD and glaucoma, we conducted a comprehensive analysis of their genetic associations using LDSR, PRS, and MR methods. Our results provide nuanced insights into the potential genetic relationships between CKD and glaucoma. Although the lack of significant genetic associations identified by LDSR suggests that shared polygenic heritability between these conditions may be limited, the significant association observed in the PRS analysis between glaucoma PRS and higher eGFR raises intriguing possibilities. This result could indicate an underlying genetic influence linking glaucoma-related genetic variants with renal function markers, specifically eGFR. However, the absence of corroborative evidence from MR analysis weakens the causal interpretation of this association and highlights the complexity of disentangling pleiotropic effects from true causal pathways. These discrepancies across methodologies underscore the importance of integrating multiple approaches when investigating shared genetic etiologies.^35^^,^^36^ It is also possible that the observed glaucoma PRS-eGFR association reflects indirect genetic influences (for example, via cilia-related genes) or interactions with unmeasured environmental or phenotypic factors. Future research using larger cohorts, more refined phenotyping, and advanced statistical techniques may help clarify these findings and determine whether the PRS signal represents a true shared genetic basis or spurious association. Additionally, exploring tissue-specific gene expression and pathway analyses could provide further insights into potential biological mechanisms linking glaucoma and renal function.

A major strength of our study lies in the utilization of two large, population-based biobanks, the Lifelines Cohort and the UK Biobank, which enhances the generalizability and robustness of our findings. Additionally, we used the SBayesRC method to calculate PRS, a state-of-the-art approach that integrates GWAS summary statistics with functional genomic annotations.^23^ Nevertheless, our study has several limitations. First, the definitions of glaucoma differed between the Lifelines and UK Biobank datasets due to differences in study design, which may introduce heterogeneity. In particular, the algorithm used in the Lifelines cohort, which prioritized specificity, resulted in a small number of “definite” cases based solely on self-reported surgery. Additionally, the lack of medical record validation for all cases poses a risk of misclassification bias. Second, in the Lifelines cohort, we were unable to distinguish between POAG and PACG. However, given the prevalence ratio of POAG to PACG in Europeans, it is likely that the majority of cases were POAG.^15^ In a subset of Lifelines, EyeLife,^20^ the ratio of POAG (combined definite and probable glaucoma) to PACG was 10:1.^37^ Consequently, we expect that any bias introduced by the inclusion of a small number of PACG cases in the Lifelines study only would be minimal and would not account for the associations we observed with eGFR. Third, due to the cross-sectional nature of our study design, the temporal sequence between kidney function and glaucoma cannot be established. Although we observed an association between higher eGFR and glaucoma, it remains uncertain whether alterations in kidney function precede the onset of glaucoma or vice versa. Finally, the restriction of our analysis to individuals of European ancestry aged 55 years and older limits the generalizability of our findings to other ethnicities and younger age groups. These limitations should be considered when interpreting the findings, and future studies with a broader age range, and more consistent phenotyping and granular data are warranted to validate our results.

CKD represents a systemic condition with a contentious relationship to POAG, reflecting the broader challenge of understanding shared mechanisms between systemic and ocular diseases. Our study contributes to resolving this controversy by leveraging large-scale population-based biobanks to provide a nuanced exploration of phenotypic and genetic links. Our findings suggest that, although CKD itself may not directly contribute to the odds of POAG, there is a notable association between a high eGFR and increased glaucoma odds. This challenges previous reports that primarily linked low eGFR to glaucoma odds and suggests the need for further investigation into the underlying biological mechanisms. Clinically, this could shift the way healthcare providers view kidney function in relation to glaucoma risk, suggesting that a high eGFR should also be considered as a potential risk factor for glaucoma, particularly in populations with preserved renal function. Additionally, the relationship between a high eGFR and glaucoma suggests that clinicians may need to reassess the implications of kidney function in the broader context of systemic health, as it may reflect subtle shifts in underlying systemic health or vascular integrity that affect the optic nerve. From a preventive standpoint, understanding the nuances of this association may guide clinicians to identify individuals with a high eGFR who could be at increased risk for glaucoma, allowing for earlier and more proactive eye health monitoring in these populations. Our genetic analysis also provides insights into potential shared pathways between renal function and POAG, contributing to a deeper understanding of their complex interplay. Future research integrating genetic and clinical data may help refine risk assessment and improve patient management at the intersection of these two diseases.

## Conclusions

In summary, our study found no evidence supporting an association between CKD or lower eGFR and glaucoma, challenging the previously presumed link between impaired renal function and glaucoma. Although we observed a statistical association with higher eGFR, the cross-sectional nature of our study precludes causal inference, and the modest effect size suggests this relationship requires further validation. Future longitudinal studies are warranted to determine whether this observation represents a true biological phenomenon or a chance finding, and to elucidate its potential underlying mechanisms.

## Supplementary Material

Supplement 1
