# Supplementary material for: Association Between Chronic Kidney Disease and Glaucoma: Results From the Lifelines Cohort Study and UK Biobank
Source: Invest Ophthalmol Vis Sci. 2025 Dec 3;66(15):17. doi: 10.1167/iovs.66.15.17 (PMC12697689; doi:10.1167/iovs.66.15.17)
Supplement: Supplement 1 [file iovs-66-15-17_s001.pdf]

eTable 1 Definitions of covariates

eTable 2 GWAS summary data used in the linkage disequilibrium score regression analysis

eTable 3 Associations of estimated glomerular filtration rate (eGFR, per 10 ml/min/1.73m^2 increase) and chronic kidney disease (CKD) with glaucoma

eTable 4 Odds ratios of glaucoma in estimated glomerular filtration rate (eGFR) quintiles compared to the middle (third) quintile

eTable 5 Associations of estimated glomerular filtration rate (eGFR, per 10 ml/min/1.73m^2 increase) and chronic kidney disease (CKD) with glaucoma in participants with diabetes

eTable 6 Odds ratios of glaucoma in estimated glomerular filtration rate (eGFR) quintiles compared to the middle (third) quintile in participants with diabetes

eTable 7 Associations of estimated glomerular filtration rate (eGFR, per 10 ml/min/1.73m^2 increase) and chronic kidney disease (CKD) with glaucoma in participants without diabetes

eTable 8 Odds ratios of glaucoma in estimated glomerular filtration rate (eGFR) quintiles compared to the middle (third) quintile in participants without diabetes

eTable 9 Associations of chronic kidney disease (CKD), estimated glomerular filtration rate (eGFR) and glaucoma with their respective polygenic risk scores (PRS, per 1 standard deviation increase)

eTable 10 Associations of polygenic risk scores of chronic kidney disease (per 1 standard deviation increase) with glaucoma

eTable 11 Odds ratios of glaucoma in chronic kidney disease (CKD) polygenic risk scores (PRS) quintiles compared to the first quintile

eTable 12 Odds ratios of chronic kidney disease (CKD) in glaucoma polygenic risk scores (PRS) quintiles compared to the first quintile

eTable 13 Associations of polygenic risk scores of estimated glomerular filtration rate (per 1 standard deviation increase) with glaucoma

eTable 14 Odds ratios of glaucoma in estimated glomerular filtration rate (eGFR) polygenic risk scores (PRS) quintiles compared to the middle (third) quintile

eTable 15 Two-sample Mendelian randomization results for the bidirectional relationships between glaucoma and estimated glomerular filtration rate (eGFR)

eFigure 1 Directed acyclic graph.

eFigure 2 Participation flow chart in the Lifelines Cohort (left) and UK Biobank (right).

eTable 1 Definitions of covariates

**Covariates**	**Definitions**
Centered age squared	Calculated as $(\text{age-mean age})^2$
Body mass index	Calculated as weight/height^2 (kg/m^2)
Hypertension	Systolic blood pressure ≥140 mmHg or diastolic blood pressure ≥90 mmHg or use of antihypertensive medication.
Diabetes	Self-reported diabetes mellitus or use of antidiabetic medication(s) or a fasting blood glucose ≥7.0 mmol/L or HbA1c ≥6.5%
Hyperlipidemia	A total cholesterol of ≥6.2 mmol/L or self-reported use of lipid-lowering drugs
Education level	Categorized as high (university education and higher vocational education in Lifelines; college or university degree in UK Biobank), middle (secondary vocational education, senior general secondary education, and pre-university secondary education in Lifelines; A levels/AS levels or equivalent, NVQ or HND or HNC or equivalent, and other professional qualifications in UK Biobank) and low level (junior general secondary education, lower or preparatory secondary vocational education, primary education and no education in Lifelines; O levels/GCSEs or equivalent, CSEs or equivalent, and none of the above in UK Biobank)
Smoking status	Categorized as smoker (current smoker or eversmoker) and non-smoker

eTable 2 GWAS summary data used in the linkage disequilibrium score regression analysis

Traits	Unit	First author	Year	Cohort/Consortium	Sample size	Ancestry	PMID
CKD	NA	Wuttke	2019	CKDGen	480,698	European	31152163
eGFR	ml/min per 1.73 m^2	Wuttke	2019	CKDGen	567,460	European	31152163
POAG	NA	Lo Faro	2024	GBMI, IGGC	1,451,742	European	38382466
IOP	mmHg	Khawaja	2018	UKBB, IGGC, EPIC-Norfolk	139,555	European	29785010

CKD: chronic kidney disease; eGFR: estimated glomerular filtration rate; POAG: primary open angle glaucoma; IOP: intraocular pressure.

eTable 3 Associations of estimated glomerular filtration rate (eGFR, per 10 ml/min/1.73m^2 increase) and chronic kidney disease (CKD) with glaucoma

	Model 1		Model 2	
	OR (95% CI)	p	OR (95% CI)	p
**Lifelines: all cases included**				
eGFR*	1.02 (0.97-1.06)	0.48	1.02 (0.98-1.07)	0.35
CKD_{Scr}	1.27 (1.02-1.57)	0.03	1.21 (0.98-1.50)	0.08
CKD_{UAE}	1.14 (0.82-1.58)	0.43	1.09 (0.79-1.51)	0.61
CKD_{UACR}	1.07 (0.74-1.55)	0.73	1.02 (0.70-1.49)	0.91
$CKD_{Scr+UAE}$	1.10 (0.85-1.43)	0.46	1.07 (0.82-1.39)	0.64
$CKD_{Scr+UACR}$	1.11 (0.84-1.47)	0.45	1.08 (0.82-1.42)	0.60
**Lifelines: possible cases excluded**				
eGFR*	1.06 (0.96-1.18)	0.25	1.06 (0.96-1.18)	0.27
CKD_{Scr}	1.01 (0.61-1.66)	0.98	0.97 (0.59-1.61)	0.92

CKD_{UAE}	1.29 (0.66-2.54)	0.45	1.22 (0.62-2.40)	0.57
CKD_{UACR}	1.13 (0.51-2.48)	0.77	1.06 (0.48-2.33)	0.89
	Model 1		Model 2	
	OR (95% CI)	p	OR (95% CI)	p
$CKD_{Scr+UAE}$	1.28 (0.74-2.21)	0.37	1.24 (0.71-2.15)	0.45
$CKD_{Scr+UACR}$	1.15 (0.64-2.08)	0.64	1.11 (0.61-2.01)	0.73
**UK Biobank: all cases included**				
eGFR*	**1.04 (1.02-1.07)**	**0.001**	**1.04 (1.02-1.07)**	**0.001**
CKD_{Scr}	1.01 (0.88-1.17)	0.86	0.97 (0.84-1.12)	0.71
**UK Biobank: possible cases excluded**				
eGFR*	**1.04 (1.01-1.07)**	**0.002**	**1.04 (1.02-1.07)**	**0.001**
CKD_{Scr}	1.01 (0.86-1.17)	0.94	0.96 (0.83-1.12)	0.63

Model 1: adjustment for age, centered age squared, and gender; Model 2: adjustment for age, centered age squared, gender, body mass index, hypertension, diabetes, hyperlipidemia, education level, and smoking status; CKD_{Scr}: eGFR <60 mL/min/1.73 m^2; CKD_{UAE}: urinary albumin excretion ≥30 mg/d; CKD_{UACR}: urinary albumin-creatinine ratio ≥30 mg/g; $CKD_{Scr+UAE}$: eGFR <60 mL/min/1.73 m^2 and/or urinary albumin excretion ≥30 mg/d; $CKD_{Scr+UACR}$: eGFR <60 mL/min/1.73 m^2 and/or urinary albumin-creatinine ratio ≥30 mg/g.
* per 10 ml/min/1.73m^2 increase in eGFR.

eTable 4 Odds ratios of glaucoma in estimated glomerular filtration rate (eGFR) quintiles compared to the middle (third) quintile

		Model 1		Model 2	
eGFR quintiles	n (glaucoma cases/controls)	OR (95% CI)	p	OR (95% CI)	p
**Lifelines: all cases included**					
quintile 1	403/3,901	0.99 (0.85-1.15)	0.91	0.97 (0.83-1.13)	0.68
quintile 2	345/3,950	0.94 (0.81-1.10)	0.45	0.94 (0.80-1.09)	0.40
quintile 3	358/3,951	reference			
quintile 4	332/3,969	0.95 (0.81-1.11)	0.50	0.94 (0.81-1.10)	0.46
quintile 5	323/3,943	1.12 (0.95-1.32)	0.18	1.10 (0.93-1.29)	0.26
**Lifelines: possible cases excluded**					
quintile 1	71/3,936	0.98 (0.68-1.39)	0.89	0.97 (0.68-1.39)	0.87
quintile 2	61/3,953	1.03 (0.72-1.49)	0.86	1.03 (0.71-1.48)	0.88

quintile 3	57/3,934	reference			
quintile 4	35/3,966	0.66 (0.43-1.01)	0.05	0.65 (0.42-0.99)	0.05
quintile 5	60/3,925	1.64 (1.11-2.41)	0.01	1.58 (1.07-2.33)	0.02
		Model 1		Model 2	
eGFR quintiles	n (glaucoma cases/controls)	OR (95% CI)	p	OR (95% CI)	p
**UK Biobank: all cases included**					
quintile 1	994/17,033	0.93 (0.85-1.02)	0.13	0.92 (0.84-1.00)	0.06
quintile 2	10,18/17,013	1.01 (0.93-1.11)	0.76	1.01 (0.93-1.11)	0.77
quintile 3	10,60/16,964	reference			
quintile 4	963/17,068	1.00 (0.91-1.09)	0.95	0.99 (0.91-1.09)	0.85
quintile 5	919/17,101	1.16 (1.05-1.27)	0.003	1.14 (1.03-1.25)	0.01
**UK Biobank: possible cases excluded**					
quintile 1	901/17,046	0.93 (0.85-1.02)	0.12	0.91 (0.83-1.00)	0.06
quintile 2	924/17,016	1.01 (0.92-1.11)	0.85	1.01 (0.92-1.11)	0.86

quintile 3	966/16,965	reference			
quintile 4	876/17,057	0.99 (0.90-1.09)	0.91	0.99 (0.90-1.09)	0.82
quintile 5	838/17,095	1.15 (1.04-1.27)	0.01	1.13 (1.02-1.25)	0.02

Model 1: adjustment for age, centered age squared, and gender;

Model 2: adjustment for age, centered age squared, gender, body mass index, hypertension, diabetes, hyperlipidemia, education level, and smoking status.

eTable 5 Associations of estimated glomerular filtration rate (eGFR, per 10 ml/min/1.73m² increase) and chronic kidney disease (CKD) with glaucoma in participants with diabetes

	Model 1		Model 2	
	OR (95% CI)	p	OR (95% CI)	p
**Lifelines: all cases included**				
eGFR*	1.02 (0.91-1.15)	0.71	1.02 (0.91-1.15)	0.71
CKD	1.38 (0.82-2.30)	0.22	1.38 (0.82-2.32)	0.22
**Lifelines: possible cases excluded**				
eGFR*	1.24 (0.95-1.62)	0.12	1.24 (0.94-1.62)	0.12
CKD	1.16 (0.41-3.25)	0.78	1.22 (0.43-3.46)	0.70
**UK Biobank: all cases included**				
eGFR*	1.02 (0.97-1.08)	0.49	1.02 (0.97-1.08)	0.38

CKD	0.97 (0.72-1.30)	0.84	0.94 (0.70-1.27)	0.70
**UK Biobank: possible cases excluded**				
eGFR*	1.01 (0.95-1.06)	0.81	1.01 (0.96-1.07)	0.63
CKD	1.01 (0.75-1.37)	0.94	0.98 (0.72-1.32)	0.89

Model 1: adjustment for age, centered age squared, and gender; Model 2: adjustment for age, centered age squared, gender, body mass index, hypertension, hyperlipidemia, education level, and smoking status; CKD: eGFR <60 mL/min/1.73 m^2.

* per 10 ml/min/1.73m^2 increase in eGFR.

eTable 6 Odds ratios of glaucoma in estimated glomerular filtration rate (eGFR) quintiles compared to the middle (third) quintile in participants with diabetes

		Model 1		Model 2	
eGFR quintiles	n (glaucoma cases/controls)	OR (95% CI)	p	OR (95% CI)	p
**Lifelines: all cases included**					
quintile 1	41/287	0.86 (0.54-1.38)	0.54	0.85 (0.53-1.37)	0.51
quintile 2	41/287	0.88 (0.56-1.40)	0.60	0.88 (0.56-1.41)	0.60
quintile 3	43/283	reference			
quintile 4	31/296	0.70 (0.43-1.14)	0.15	0.70 (0.43-1.15)	0.16
quintile 5	44/284	1.10 (0.68-1.78)	0.71	1.08 (0.66-1.75)	0.77
**Lifelines: possible cases excluded**					

quintile 1	8/288	0.83 (0.29-2.39)	0.73	0.83 (0.29-2.40)	0.73
quintile 2	9/286	1.13 (0.41-3.11)	0.82	1.12 (0.40-3.10)	0.83
quintile 3	7/289	reference			
quintile 4	2/293	0.34 (0.07-1.65)	0.18	0.34 (0.07-1.68)	0.19
quintile 5	14/281	**4.04 (1.45-11.26)**	**0.007**	**3.90 (1.39-10.89)**	**0.009**
		Model 1		Model 2	
eGFR quintiles	n (glaucoma cases/controls)	OR (95% CI)	p	OR (95% CI)	p
**UK Biobank: all cases included**					
quintile 1	125/1,596	0.91 (0.71-1.17)	0.47	0.88 (0.68-1.14)	0.33
quintile 2	135/1,588	1.03 (0.80-1.32)	0.81	1.02 (0.79-1.31)	0.90
quintile 3	136/1,581	reference			
quintile 4	118/1,602	0.97 (0.75-1.26)	0.82	0.96 (0.74-1.24)	0.74
quintile 5	111/1,609	0.98 (0.74-1.29)	0.87	0.96 (0.73-1.27)	0.76
**UK Biobank: possible cases excluded**					

quintile 1	115/1,595	0.91 (0.70-1.18)	0.46	0.87 (0.67-1.14)	0.32
quintile 2	123/1,589	1.01 (0.78-1.31)	0.92	1.00 (0.77-1.29)	0.99
quintile 3	126/1,581	reference			
quintile 4	112/1,597	0.99 (0.75-1.29)	0.93	0.98 (0.75-1.28)	0.86
quintile 5	96/1,614	0.88 (0.66-1.18)	0.39	0.86 (0.64-1.16)	0.32

Model 1: adjustment for age, centered age squared, and gender;

Model 2: adjustment for age, centered age squared, gender, body mass index, hypertension, hyperlipidemia, education level, and smoking status.

eTable 7 Associations of estimated glomerular filtration rate (eGFR, per 10 ml/min/1.73m^2 increase) and chronic kidney disease (CKD) with glaucoma in participants without diabetes

	Model 1		Model 2	
	OR (95% CI)	p	OR (95% CI)	p
**Lifelines: all cases included**				
eGFR*	1.01 (0.97-1.06)	0.63	1.02 (0.98-1.07)	0.45
CKD	1.22 (0.97-1.55)	0.09	1.20 (0.95-2.52)	0.13
**Lifelines: possible cases excluded**				
eGFR*	1.03 (0.92-1.15)	0.61	1.03 (0.92-1.15)	0.62
CKD	0.92 (0.51-1.65)	0.78	0.92 (0.52-1.66)	0.79

**UK Biobank: all cases included**				
eGFR*	**1.05 (1.02-1.07)**	**0.0009**	**1.05 (1.02-1.08)**	**0.0008**
CKD	0.99 (0.84-1.17)	0.89	0.98 (0.83-1.16)	0.83
**UK Biobank: possible cases excluded**				
eGFR*	**1.05 (1.02-1.08)**	**0.0007**	**1.05 (1.02-1.08)**	**0.0007**
CKD	0.96 (0.81-1.15)	0.68	0.96 (0.80-1.14)	0.63

Model 1: adjustment for age, centered age squared, and gender; Model 2: adjustment for age, centered age squared, gender, body mass index, hypertension, hyperlipidemia, education level, and smoking status; CKD: eGFR <60 mL/min/1.73 m^2.

* per 10 ml/min/1.73m^2 increase in eGFR.

eTable 8 Odds ratios of glaucoma in estimated glomerular filtration rate (eGFR) quintiles compared to the middle (third) quintile in participants without diabetes

		Model 1		Model 2	
eGFR quintiles	n (glaucoma cases/controls)	OR (95% CI)	p	OR (95% CI)	p
**Lifelines: all cases included**					
quintile 1	360/3,608	1.01 (0.86-1.18)	0.93	0.99 (0.84-1.17)	0.91
quintile 2	305/3,682	0.94 (0.79-1.10)	0.44	0.93 (0.79-1.10)	0.40
quintile 3	315/3,634	reference			
quintile 4	303/3,668	0.98 (0.83-1.16)	0.82	0.99 (0.83-1.16)	0.86
quintile 5	278/3,685	1.08 (0.91-1.29)	0.39	1.08 (0.91-1.29)	0.37

**Lifelines: possible cases excluded**					
quintile 1	63/3,647	1.01 (0.69-1.48)	0.96	1.02 (0.69-1.49)	0.94
quintile 2	52/3,656	1.01 (0.68-1.49)	0.97	1.01 (0.68-1.49)	0.97
quintile 3	50/3,658	reference			
quintile 4	33/3,663	0.70 (0.45-1.09)	0.12	0.70 (0.45-1.10)	0.12
quintile 5	46/3,653	1.36 (0.89-2.08)	0.16	1.36 (0.89-2.08)	0.16
		Model 1		Model 2	
eGFR quintiles	n (glaucoma cases/controls)	OR (95% CI)	p	OR (95% CI)	p
**UK Biobank: all cases included**					
quintile 1	864/15,445	0.93 (0.85-1.03)	0.15	0.93 (0.84-1.02)	0.13
quintile 2	888/15,425	1.01 (0.92-1.11)	0.79	1.01 (0.92-1.11)	0.82
quintile 3	924/15,375	reference			
quintile 4	852/15,464	1.01 (0.91-1.11)	0.89	1.01 (0.91-1.11)	0.91
quintile 5	**801/15,494**	**1.17 (1.05-1.29)**	**0.003**	**1.16 (1.05-1.29)**	**0.004**

**UK Biobank: possible cases excluded**					
quintile 1	783/15,450	0.93 (0.84-1.02)	0.14	0.92 (0.83-1.02)	0.12
quintile 2	804/15,420	1.01 (0.91-1.11)	0.90	1.00 (0.91-1.11)	0.92
quintile 3	842/15,386	reference			
quintile 4	769/15,456	1.00 (0.90-1.10)	0.95	1.00 (0.90-1.10)	0.93
quintile 5	**735/15,491**	**1.17 (1.05-1.30)**	**0.004**	**1.17 (1.05-1.30)**	**0.005**

Model 1: adjustment for age, centered age squared, and gender;

Model 2: adjustment for age, centered age squared, gender, body mass index, hypertension, hyperlipidemia, education level, and smoking status.

eTable 9 Associations of chronic kidney disease (CKD), estimated glomerular filtration rate (eGFR) and glaucoma with their respective polygenic risk scores (PRS, per 1 standard deviation increase)

	OR / b	95% CI	p
Lifelines Cohort			
CKD vs CKD PRS	1.99	1.80-2.19	$< 2\times10^{-16}$
eGFR vs eGFR PRS	4.59	4.40-4.77	$< 2\times10^{-16}$
glaucoma vs glaucoma PRS	1.38	1.29-1.47	$< 2\times10^{-16}$
UK Biobank			

CKD vs CKD PRS	1.79	1.70-1.89	$< 2\times10^{-16}$
eGFR vs eGFR PRS	3.92	3.85-4.00	$< 2\times10^{-16}$
glaucoma vs glaucoma PRS	1.56	1.51-1.60	$< 2\times10^{-16}$

eTable 10 Associations of polygenic risk scores of chronic kidney disease (per 1 standard deviation increase) with glaucoma

	n (glaucoma cases/controls)	OR (95% CI)	p
Lifelines: all cases included	942/10,686	1.04 (0.97-1.11)	0.25
Lifelines: possible cases excluded	153/10,686	1.01 (0.86-1.19)	0.89
UK Biobank: all cases included	4,891/83,731	0.98 (0.94-1.02)	0.30
UK Biobank: possible cases excluded	4,445/83,731	0.98 (0.94-1.02)	0.36

eTable 11 Odds ratios of glaucoma in chronic kidney disease (CKD) polygenic risk scores (PRS) quintiles compared to the first quintile

CKD PRS quintiles	n (glaucoma cases/controls)	OR (95% CI)	p
**Lifelines: all cases included**			
quintile 1	185/2,141	reference	
quintile 2	176/2,149	0.95 (0.77-1.19)	0.68

quintile 3	190/2,136	1.03 (0.84-1.28)	0.77
quintile 4	199/2,126	1.07 (0.87-1.32)	0.51
quintile 5	192/2,134	1.04 (0.84-1.28)	0.72
**Lifelines: possible cases excluded**			
quintile 1	31/2,137	reference	
quintile 2	20/2,148	0.66 (0.37-1.16)	0.14
quintile 3	39/2,128	1.27 (0.79-2.05)	0.33
quintile 4	34/2,134	1.09 (0.67-1.78)	0.73
quintile 5	29/2,139	0.93 (0.56-1.55)	0.78
CKD PRS quintiles	n (glaucoma cases/controls)	OR (95% CI)	p
**UK Biobank: all cases included**			
quintile 1	976/16,749	reference	
quintile 2	987/16,737	1.01 (0.92-1.11)	0.82
quintile 3	963/16,761	0.98 (0.90-1.08)	0.71

quintile 4	1,021/16,703	1.04 (0.95-1.14)	0.36
quintile 5	944/16,781	0.92 (0.84-1.02)	0.10
**UK Biobank: possible cases excluded**			
quintile 1	887/16,749	reference	
quintile 2	894/16,741	1.01 (0.91-1.11)	0.90
quintile 3	872/16,763	0.98 (0.89-1.08)	0.67
quintile 4	929/16,706	1.04 (0.95-1.15)	0.37
quintile 5	863/16,772	0.93 (0.84-1.02)	0.13

eTable 12 Odds ratios of chronic kidney disease (CKD) in glaucoma polygenic risk scores (PRS) quintiles compared to the first quintile

Glaucoma PRS quintiles	n (CKD cases/controls)	OR (95% CI)	p
**Lifelines**			

quintile 1	101/2,225	reference	
quintile 2	93/2,232	0.95 (0.71-1.27)	0.72
quintile 3	86/2,240	0.88 (0.65-1.19)	0.41
quintile 4	104/2,221	1.08 (0.81-1.44)	0.61
quintile 5	92/2,234	0.95 (0.70-1.27)	0.72
**UK Biobank**			
quintile 1	643/17,082	reference	
quintile 2	617/17,107	0.95 (0.84-1.06)	0.34
quintile 3	699/17,025	1.07 (0.96-1.19)	0.23
quintile 4	661/17,063	1.00 (0.89-1.12)	0.99
quintile 5	645/17,080	0.96 (0.85-1.07)	0.44

eTable 13 Associations of polygenic risk scores of estimated glomerular filtration rate (per 1 standard deviation increase) with glaucoma

	n (glaucoma cases/controls)	OR (95% CI)	p
Lifelines: all cases included	942/10,686	1.05 (0.98-1.12)	0.13

Lifelines: possible cases excluded	153/10,686	1.02 (0.87-1.20)	0.79
UK Biobank: all cases included	4,891/83,731	1.01 (0.98-1.04)	0.62
UK Biobank: possible cases excluded	4,445/83,731	1.01 (0.97-1.04)	0.74

eTable 14 Odds ratios of glaucoma in estimated glomerular filtration rate (eGFR) polygenic risk scores (PRS) quintiles compared to the middle (third) quintile

eGFR PRS quintiles	n (glaucoma cases/controls)	OR (95% CI)	p
**Lifelines: all cases included**			

quintile 1	175/2,151	1.00 (0.80-1.25)	0.99
quintile 2	195/2,130	1.12 (0.90-1.38)	0.32
quintile 3	174/2,152	reference	
quintile 4	193/2,132	1.13 (0.91-1.40)	0.28
quintile 5	205/2,121	1.18 (0.95-1.45)	0.13
**Lifelines: possible cases excluded**			
quintile 1	34/2,134	1.05 (0.65-1.72)	0.83
quintile 2	22/2,146	0.67 (0.39-1.16)	0.16
quintile 3	32/2,135	reference	
quintile 4	31/2,137	0.98 (0.60-1.62)	0.94
quintile 5	34/2,134	1.04 (0.64-1.69)	0.88
eGFR PRS quintiles	n (glaucoma cases/controls)	OR (95% CI)	p
**UK Biobank: all cases included**			
quintile 1	982/16,743	0.98 (0.90-1.08)	0.70

quintile 2	980/16,744	0.98 (0.90-1.07)	0.67
quintile 3	998/16,726	reference	
quintile 4	953/16,771	0.95 (0.87-1.04)	0.31
quintile 5	978/16,747	0.99 (0.90-1.08)	0.77
**UK Biobank: possible cases excluded**			
quintile 1	905/16,731	1.01 (0.92-1.11)	0.78
quintile 2	889/16,746	0.99 (0.90-1.09)	0.92
quintile 3	893/16,742	reference	
quintile 4	873/16,762	0.98 (0.89-1.08)	0.64
quintile 5	885/16,750	1.00 (0.91-1.10)	0.98

eTable 15 Two-sample Mendelian randomization results for the bidirectional relationships between glaucoma and estimated glomerular filtration rate (eGFR)

No. of SNPs	Exposure	Outcome	Methods	Beta (95% CI)	p	Heterogeneity statistic	p	Pleiotropy statistic	p
152	eGFR	Glaucoma	Inverse variance weighted	0.090 (-0.61~0.79)	0.80	Q=269.00	1.16×10^{-8}	NA	NA
152	eGFR	Glaucoma	MR-Egger	0.44 (-1.20~2.08)	0.60	Q=268.62	9.32×10^{-9}	Intercept=-0.0014	0.65
152	eGFR	Glaucoma	Weighted median	-0.059 (-0.92~0.80)	0.89	NA	NA	NA	NA
76	Glaucoma	eGFR	Inverse variance weighted	0.00089 (-0.0010~0.0028)	0.36	Q=278.56	3.47×10^{-25}	NA	NA
76	Glaucoma	eGFR	MR-Egger	-0.0026 (-0.0080~0.0027)	0.34	Q=271.66	2.29×10^{-24}	Intercept=0.00034	0.17
76	Glaucoma	eGFR	Weighted median	0.00073 (-0.0010~0.0024)	0.40	NA	NA	NA	NA

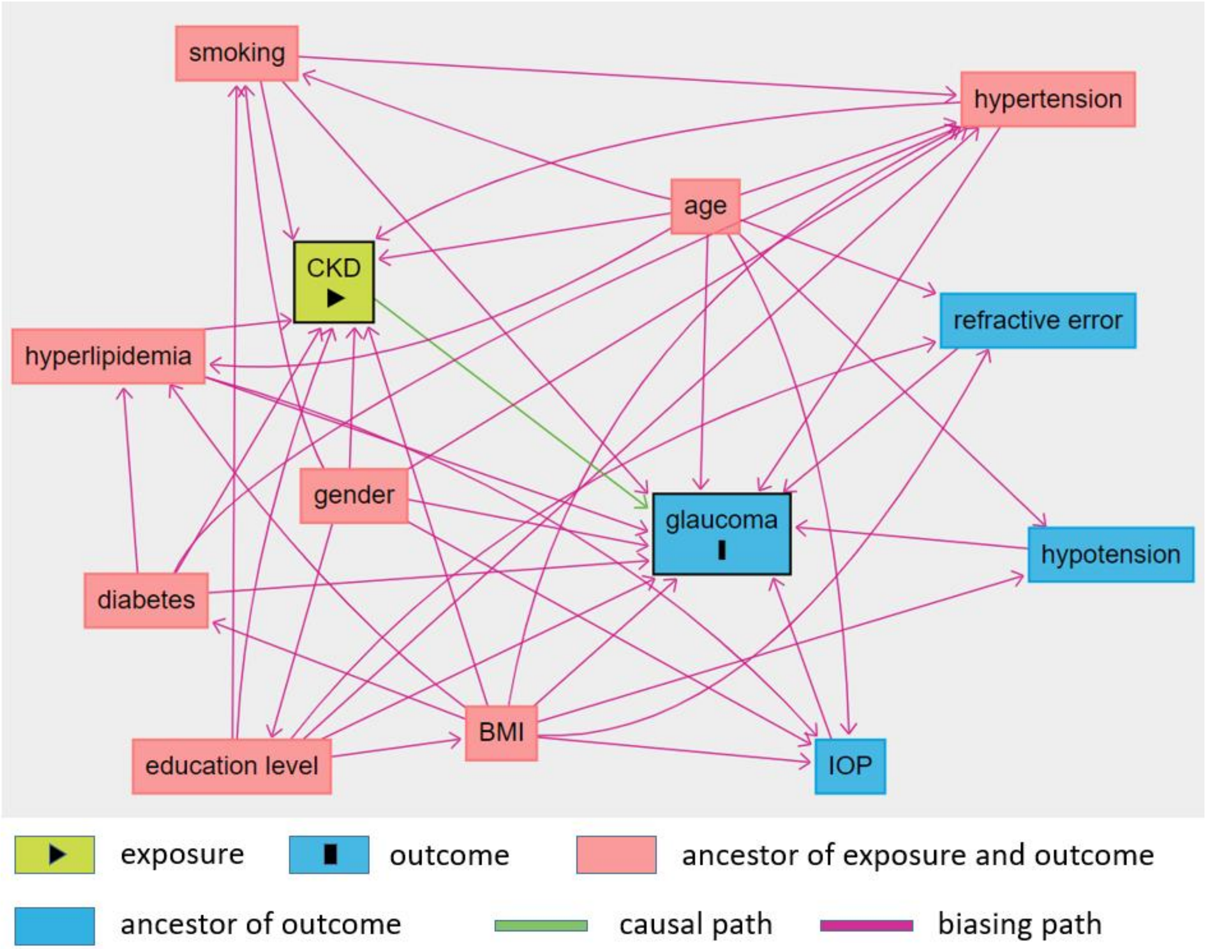

eFigure 1 Directed acyclic graph. CKD: chronic kidney disease; IOP: intraocular pressure; BMI: body mass index.

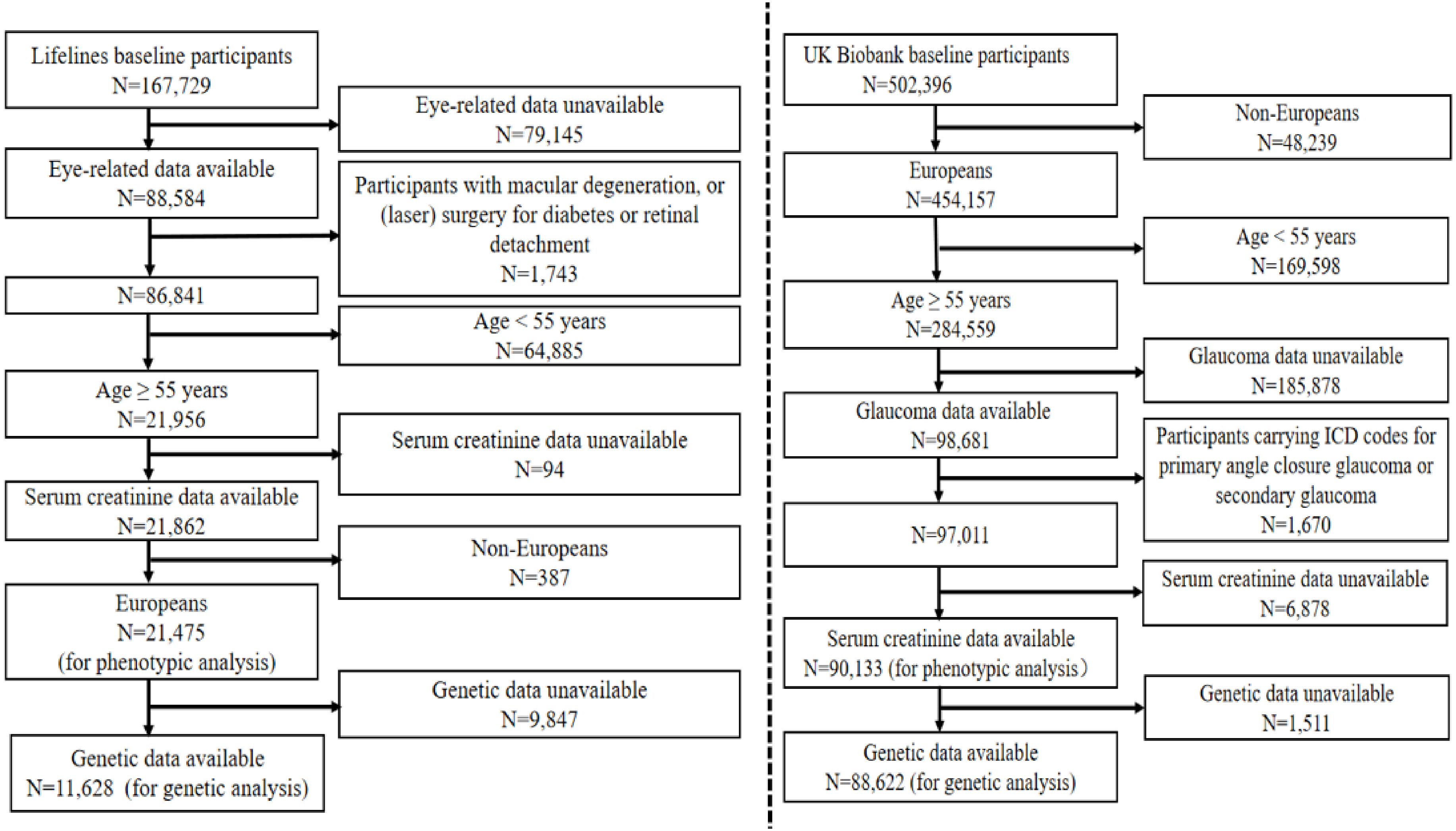

eFigure 2 Participation flow chart in the Lifelines Cohort (left) and UK Biobank (right).
